# The correlation between vitamin a status and refractory *Mycoplasma Pneumoniae* pneumonia (RMPP) incidence in children

**DOI:** 10.1186/s12887-020-02254-y

**Published:** 2020-07-30

**Authors:** Yuanyuan Li, Ziyao Guo, Guangli Zhang, Xiaoyin Tian, Qinyuan Li, Dapeng Chen, Zhengxiu Luo

**Affiliations:** 1 Chongqing Key Laboratory of Pediatrics, China International Science and Technology Cooperation base of Child Development and Critical Disorders, Chongqing, 400014 China; 2 Key Laboratory of Child Development and Disorders; National Clinical Research Center for Child Health and Disorders, Department of Children’s Hospital of Chongqing Medical University of Education, Chongqing, 400014 China; 3 Department of Respiratory Medicine Children’s Hospital of Chongqing Medical University, National Clinical Research Center for Child Health and Disorders, Ministry of Education Key Laboratory of Child Development and Disorders, Chongqing, 400014 China; 4 Department of Clinical Laboratory center, Children’s Hospital of Chongqing Medical University, Chongqing, 400014 China

**Keywords:** Vitamin a, Retinol-binding protein (RBP), *Mycoplasma Pneumoniae* pneumonia (MPP)

## Abstract

**Background:**

Vitamin A plays a pivotal role in respiratory infection, accurate estimation of vitamin A status was recommended in planning and implementing interventions. As infections affect serum vitamin A productions, the real status need to be adjusted by acute phase protein (APP). *Mycoplasma pneumoniae* is an important cause of respiratory infection in children, the association between vitamin A concentrations and refractory *Mycoplasma pneumoniae* pneumonia (RMPP) remains unclear.

**Methods:**

181 MPP patients were enrolled in this retrospective study, adjusted vitamin A concentrations and other parameters were compared between RMPP and general-MPP (GMPP) patients. Multivariate logistic regression test was performed to evaluate the association between vitamin A levels and RMPP incidence, linear correlation tests were applied to evaluate correlation between vitamin A concentrations and fever duration, length of stay (LOS).

**Results:**

Vitamin A concentrations in RMPP group were significantly lower than those in GMPP patients (*P* < 0.05), vitamin A (OR = 0.795, 95% C. I 0.669–0.946) and CRP (OR = 1.050, 95% C. I 1.014–1.087) were independently associated with RMPP incidence. Linear correlation tests found vitamin A concentrations were negatively correlated with fever duration and LOS (*P* < 0.001).

**Conclusions:**

Serum vitamin A concentrations were independently associated with RMPP incidence, which may correlate with reduced incidence of RMPP.

## Background

*Mycoplasma pneumoniae (M. pneumoniae)* is the predominant pathogen of community-acquired pneumonia (CAP), which contributes to approximately 10 to 40% of CAP cases in children [1–3]. Most pediatric cases of *M. pneumoniae* pneumonia (MPP) are benign and self-limited, however, there still are some cases showing clinical and radiological deterioration despite of macrolides antibiotic therapy for 7 days or longer, which are defined as refractory *Mycoplasma pneumoniae* pneumonia (RMPP) [4, 5]. The exact mechanisms of RMPP are not fully clarified, reducing the incidence of RMPP and improving its prognosis remain challenges. Our previous study and other literatures demonstrated glucocorticoid therapy attenuated the clinical manifestations, radiological findings and length of stay (LOS) of RMPP children [6, 7], indicating excessive inflammation involved in RMPP pathogenesis [8].

Micronutrients share inter-dependent relationships with host’s infection immunity [9, 10]. As acute infection affects concentrations of some micronutrients (including vitamin A, ferritin) [11–13], those circulating concentrations should be adjusted by acute phase protein (APP) to eliminate the impact of infection and reflect real micronutrient status [13–15]. The most two commonly used APPs are C-reactive protein (CRP) and α-1-acid glycoprotein (AGP) [14, 15]. Vitamin A is an essential micronutrient governs broad range of biological processes [16]. Recent studies highlight the interactions between vitamin A status and immune response [9, 17], demonstrating vitamin A deficiency (VAD) may cause imbalance between pro- and anti-inflammatory factors and excessive immune response [18], which emerged in RMPP. Serum retinol or retinol-binding protein (RBP) concentrations represent vitamin A status. High-performance liquid chromatography (HPLC) is recommended for serum retinol assessment, while it’s expensive and technically challenging. The method of RBP assessment takes the advantages of being more robust for sample collection and handling processes. Therefore, RBP is often substituted as an indicator of vitamin A status. Literatures have showed higher incidence of VAD in MPP children than healthy children, and VAD was associated with MPP severity, which indicated vitamin A levels could be associated with RMPP incidence [19]. Based on all above, we hypothesis that vitamin A levels could be associated with RMPP incidence. Therefore, we constructed this retrospective study to investigate adjusted vitamin A concentrations in MPP children and clarify the association between adjusted vitamin A levels and RMPP incidence, trying to provide more evidence for RMPP intervention.

## Methods

### Study population

This study was retrospectively conducted in Children’s Hospital of Chongqing Medical University, a 1500-bed tertiary level III teaching hospital in Chongqing, China. The study was conducted in accordance with the Declaration of Helsinki and approved by the Ethics Committee of Children’s Hospital of Chongqing Medical University. Data from patients were analyzed anonymously for hospitalized *mycoplasma pneumoniae* pneumonia (MPP) children from 1 September 2018 to 31 December 2019 retrospectively. The inclusions had the following characteristics: (i) inpatients MPP children; (ii) age between 6 months and 18 years old. The exclusion criteria included any of the following: (i) patients who had an underlying organ dysfunction;(ii) patients coinfected with other pathogens. According to the diagnostic criteria of RMPP, patients were divided in to RMPP group and general *M. pneumoniae* pneumonia (GMPP) group. Besides, 65 children with micronutrients measurements in Physical Examination Center matched with age, gender and testing time were selected as the healthy control group.

### Definitions

MPP was diagnosed based on the followings: (i) clinical presentation (fever, cough, etc); (ii) chest imaging with infiltrates; (iii) having the positive results for *MP* polymerase chain reaction (PCR) tests of nasopharyngeal secretions with serum anti-*MP* IgM titer ≥1:160. The diagnosis of refractory *M. pneumoniae* pneumonia (RMPP) was based on the presence of persistent fever and clinical manifestations as well as radiological deterioration after regular macrolides treatment for 7 days or longer [4, 5], the other cases were defined as general *M. pneumoniae* pneumonia (GMPP). The body mass index (BMI) was calculated by weight in kilograms divided by height in meters squared (kg/m^2^). Extrapulmonary presentations include liver function abnormalities, myocarditis, encephalitis, rash, proteinuria, hemolytic anemia and arthritis. VAD was defined as RBP concentration lower than 0.7 μmol/L (15 mg/L) [20].

### M. pneumoniae detection

The specific antibodies against *M. pneumoniae* (IgG and IgM) were detected with passive particle agglutination (SERODIA-MYCO II, Japan) in nearly 2 ml serum samples of children on admission, MP antibody > 1:160 is a positive finding. Nasopharyngeal aspirate (NPA) was used for *M. Pneumoniae* DNA detection. In accordance with the manufacturer instructions, NPA was centrifugated 12,000 g for 5 min at 4 °C, the sediment was collected for DNA extraction with a real-time PCR commercial kit (Daan Gene Co. Ltd., Guangzhou, China). The DNA was then amplified using PCR primers and probes. Quantification curves were plotted using several concentrations of standard control samples (Daan Gene Co. Ltd., Guangzhou, China).

### Micronutrients detection

Blood samples were collected from all inpatients during the first 24 h of admission. Serum micronutrients included ferritin, vitamin A, vitamin D, folate and vitamin B12 productions. Vitamin A concentrations were measured by retinol-binding protein (RBP). Vitamin D productions were measured by 25-hydroxy vitamin D (25(OH)D). Ferritin, folate, vitamin B12 and 25(OH)D concentrations were evaluated by Chemi Luminescence (Siemens, Germany), RBP levels were evaluated by Turbidimetric inhibition immunoassay (Homa Biological, Beijing, China). For accurate estimation, vitamin A and ferritin concentrations were adjusted by CRP, using regression correction (RC) approach [14, 21].

### Adjustment approach

Adjusted vitamin A concentrations were obtained through RC approach [14, 21]. The RC approach was applied according to BRINDA methods articles, which uses linear regression to adjust RBP by the concentration of CRP. Briefly, the adjusted RBP equation was calculated by subtracting the influence of CRP, and RMPP as follows:

RBP _adjusted_ = RBP _unadjusted_ -β1 (CRP _observe_ - CRP _reference_) -β2 (RMPP),

According to available data, β1 is the CRP regression coefficient, β2 is the RMPP regression coefficient, the reference of non-logged CRP is 8 mg/L, the minimum threshold of CRP measurement. CRP and RBP are all ln transformed, CRP and RBP are continuous variables, and RMPP is a dichotomous variable. The correction was only applied to individuals with CRP > 8 mg/L to avoid over adjustment. The same approach was applied to adjust ferritin.

### BALF cytokines measurement

*Mycoplasma pneumoniae* pneumonia patients who received bronchoalveolar lavage, the bronchoalveolar lavage fluid (BALF) was extracted and collected, then delivered to the Center Laboratory Medicine immediately (no more than half an hour), kinds of cytokines including IL-2, IL-4, IL-6, IL-10, TNF-α, IFN-γ, IL-17a were measured by Cytometric Bead Array (CBA) (Cell-Gene, Hangzhou, China).

### Data collection

Demographic characteristics (age, gender, weight, BMI), extrapulmonary manifestation, serum inflammatory factors (CRP, PCT, LDH, prealbumin), BALF inflammatory cytokines (IL-2, IL-4, IL-6, IL-10, TNF-α, IFN-γ, IL-17a), serum micronutrients (ferritin, vitamin A, vitamin D, folate and vitamin B12), oxygenation, fever duration and length of stay (LOS) were retrospectively collected from all children who were included in the study.

### Statistical analysis

The continuous variables were presented as medians with 25 and 75% quartiles (interquartile range, IQR); the non-parametric test and Mann-Whitney U test were used for analysis. The categorical variables were presented as counts (percentages), and assessed by Kruskal-Wallis or the Fisher exact test. Correlations between variables were analyzed by Pearson Correlation. Univariate and multivariate logistic regression tests were used to evaluate the association between RMPP incidence and other variables. All the statistical analyses were conducted using SPSS 25.0 and Graphpad Prism 7.0 for Windows.

## Results

### Study population

**Fig. 1:**
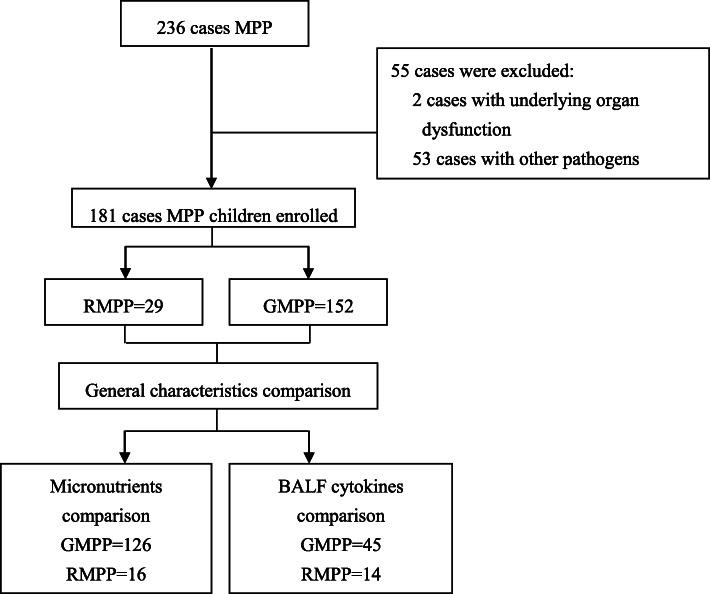
Flow diagram of the participants. As shown, a total of 291 cases met the inclusion criteria, after exclusion, 181 MPP patients were enrolled according to the inclusion and exclusion criteria, and then divided into RMPP group (*n* = 29) and GMPP group (*n* = 152). The vitamin A concentrations and other characteristics of each group were then determined

A total of 236 children diagnosed with MPP enrolled in this study, after exclusion, 181 MPP children were involved in general characteristics comparison. Among them, 142 children with micronutrients measurements were compared with micronutrient measurements of the controls. The grouped comparisons of cytokines were applied to the patients who received bronchoalveolar lavage only. The detailed breakdown of the participants is showing in Fig. 1.

### General characteristics of RMPP and GMPP patients

One hundred and eighty-one children diagnosed with MPP were divided into RMPP (*n* = 29) and GMPP (*n* = 152) according to the diagnostic criteria. No significant difference was found in age, gender and BMI between the two groups. The levels of CRP [20.00 (0.00–32.50) vs. 0 (0.00–11.00), mg/L, *P* < 0.05], PCT [0.188 (0.105–0.716) vs. 0.074 (0.043–0.132), mg/L, P < 0.05], LDH [388.00 (298.50–480.80) vs. 331.50 (288.00–381.25), U/L, *P* < 0.05] were significantly higher in RMPP than those in GMPP patients, while prealbumin productions were significantly lower in RMPP than that in GMPP patients [82.00 (68.00–100.00) vs. 115.00 (97.00–151.00), g/L, *P* < 0.05]. As expected, RMPP group had longer fever duration (10.00 (9.00–13.00) vs. 5.00 (2.00–8.00), days, P < 0.05) and length of stay (LOS) (9.00 (7.50–10.00) vs. 6.00 (5.00–7.75), days, P < 0.05), higher incidence of VAD (68.75% vs. 31.75%), extrapulmonary manifestations (58.62% vs. 14.47%) and oxygenation (100.00% vs. 45.39%) when compared to GMPP patients. (Table 1).

**Table 1:** General characteristics of MPP children

General characteristics	GMPP (n = 152)	RMPP (n = 29)	P
Age (month), Median (IQR)	46.00 (26.25–72.25)	55.00 (37.00–83.00)	0.062
Male/Female	82/70	18/11	0.422
BMI (kg/m^2^), Median (IQR)	16.44 (15.08–17.78)	15.92 (14.58–16.40)	0.168
Extrapulmonary manifestations (n, %)	22 (14.47%)	17 (58.62%)	< 0.001*
Oxygenation (n, %)^a^	69 (45.39%)	29 (100.00%)	< 0.001*
Fever duration (day), Median (IQR)	5.00 (2.00–8.00)	10.00 (9.00–13.00)	< 0.001*
CRP (mg/L), Median (IQR)^b^	0 (0.00–11.00)	20.00 (0.00–32.50)	< 0.001*
PCT (mg/L), Median (IQR)	0.074 (0.043–0.132)	0.188 (0.105–0.716)	< 0.001*
Prealbumin (g/L), Median (IQR)	115.00 (97.00–151.00)	82.00 (68.00–100.00)	< 0.001*
LDH (U/L), Median (IQR)	331.50 (288.00–381.25)	388.00 (298.50–480.80)	0.017*
LOS (day), Median (IQR)	6.00 (5.00–7.75)	9.00 (7.50–10.00)	< 0.001*
VAD (n, %)	40 (31.75%)	11 (68.75%)	0.004*

* showed difference between RMPP and GMPP groups (P < 0.05)

^a^ including nasal oxygen breath and Continuous Positive Airway Pressure (CPAP)

^b^ CRP values under minimum threshold of measurement (8 mg/L) were taken as 0 mg/L

### Serum micronutrients status in RMPP and GMPP patients

Serum micronutrients in RMPP (*n* = 16), GMPP(*n* = 126) patients and control children (*n* = 65) were presented in Table 2. Both serum unadjusted-vitamin A concentrations [10.90 (9.38–14.98) vs. 16.95 (13.38–22.63) vs. 25.10(21.90–29.05), mg/L, < 0.001] and adjusted- vitamin A [12.23(9.83–15.43) vs. 17.00(13.53–22.93) vs. 25.10(21.90–29.05), mg/L, < 0.001] in RMPP patients were significantly lower than those in healthy children and GMPP patients. Conversely, RMPP patients had remarkably higher serum unadjusted-ferritin [179.50 (109.23–290.85) vs. 95.85 (60.68–143.25) vs. 47.00 (34.55–67.10), mg/L, < 0.001] and adjusted-ferritin [171.45(104.90–238.55) vs. 96.40(61.00–143.20) vs. 47.00 (34.55–67.10), mg/L, < 0.001] productions when compared to GMPP patients and control children. No significant difference of vitamin B 12, vitamin D and folate levels was found among the three groups.

**Table 2:** Micronutrients measurements comparison of participant children

Micronutrients	Healthy control (n = 65)	GMPP (n = 126)	RMPP (n = 16)	P
Unadjusted-ferritin (mg/L), Median (IQR)	47.00 (34.55–67.10)	95.85 (60.68–143.25)	179.50 (109.23–290.85)	< 0.001*
Adjusted-ferritin (mg/L), Median (IQR) ^a^	47.00 (34.55–67.10)	96.40 (61.00–143.20)	171.45 (104.90–238.55)	< 0.001*
Vitamin B12 (pg/mL), Median (IQR)	902.00 (737.00–1031.00)	1090.00 (901.00–1604.00)	942.00 (685.00–1333.00)	0.165
Folate (ng/mL), Median (IQR)	16.45 (12.81–19.85)	16.64 (13.00–20.53)	15.87 (14.66–19.75)	0.803
Vitamin D (ng/mL), Median (IQR)	19.93 (16.28–24.06)	22.10 (15.53–31.54)	24.92 (16.54–33.94)	0.227
Unadjusted-vitamin A (mg/L), Median (IQR)	25.10 (21.90–29.05)	16.95 (13.38–22.63)	10.90 (9.38–14.98)	< 0.001*
Adjusted-vitamin A (mg/L), Median (IQR) ^a^	25.10 (21.90–29.05)	17.00 (13.53–22.93)	12.23 (9.83–15.43)	< 0.001*

* showed difference among all groups (P < 0.05)

^a^ Adjusted by CRP using the Regression Correction (RC) approach

### BALF inflammatory cytokines in RMPP and GMPP patients

The inflammatory cytokines in BALF were compared between RMPP and GMPP patients (Table 3). The levels of IL-6 [302.27 (141.45–726.08) vs. 122.00 (43.92–294.49), pg/mL, *P* < 0.05] and TNF-α [29.41 (5.01–79.13) vs. 5.56 (0.00–11.04), pg/mL, P < 0.05] were significantly higher in RMPP than those in GMPP patients. No significant differences of IL-2, IL-4, IL-10, IFN-γ, IL-17a productions was found.

**Table 3:** BALF inflammatory cytokines between GMPP and RMPP children

BALF inflammatory cytokines	GMPP (***n*** = 45)	RMPP (n = 14)	P
IL-6 (pg/mL)	122.00 (43.92–294.49)	302.27 (141.45–726.08)	0.017*
TNF-α (pg/mL)	5.56 (0.00–11.04)	29.41 (5.01–79.13)	0.014*
IL-2 (pg/mL)	0.00 (0.00–0.00)	0.00 (0.00–0.00)	0.314
IL-4 (pg/mL)	0.00 (0.00–2.06)	0.00 (0.00–0.74)	0.841
IL-10 (pg/mL)	3.09 (0.00–9.27)	9.74 (0.00–37.48)	0.227
IFN-γ (pg/mL)	3.87 (0.00–12.26)	6.74 (2.40–46.56)	0.143
IL-17a (pg/mL)	3.70 (0.00–7.76)	6.82 (0.38–10.74)	0.214

* showed difference between RMPP and GMPP groups (P < 0.05)

### Independent associated factors of RMPP

Regression analysis was used to find the associated factors of RMPP and the results were presented in Table 4. Univariate logistic regression analysis showed serum levels of adjusted-vitamin A, CRP, PCT, LDH, prealbumin and adjusted-ferritin, TNF-α productions in BALF were significantly associated with RMPP. Multivariate logistic regression analysis stated serum CRP and adjusted-vitamin A concentrations were independently associated with RMPP. Vitamin A is a protective factor, every unit decrease of adjusted-vitamin A (mg/L) resulted in 0.205 odds increase in RMPP (95% C. I 0.669–0.946); CRP is a risk factor, every unit increase of CRP (mg/L) resulted in 0.050 odds increase in RMPP (95% CI 1.014–1.087).

**Table 4:** Multivariate logistic regression analysis of RMPP

variables	Univariate		Multivariate	
	OR (95% C.I)	P	OR (95% C.I)	P
CRP	1.043 (1.021–1.065)	< 0.001	1.050 (1.014–1.087)	0.007
Adjusted-vitamin A ^a^	0.747 (0.634–0.882)	0.001	0.795 (0.669–0.946)	0.010
PCT	12.534 (3.587–43.794)	< 0.001	–	–
LDH	1.005 (1.002–1.009)	0.005	–	–
Prealbumin	0.980 (0.965–0.995)	0.011	–	–
Ferritin1	1.009 (1.003–1.016)	0.002	–	–
TNF-α	1.046 (1.016–1.077)	0.002	–	–

^a^ Adjusted by CRP using the Regression Correction (RC) approach

### Correlation between vitamin a and fever duration, LOS in MPP children

**Fig. 2:**
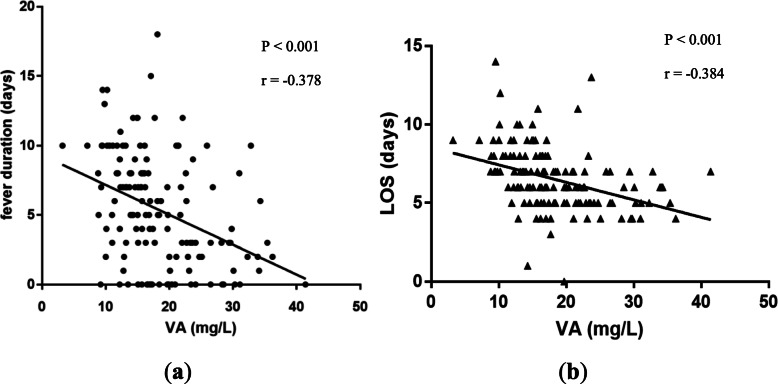
Correlation between serum vitamin A levels and clinical finding in MPP children. **a** Correlation between serum adjusted-vitamin A concentrations and fever duration in MPP children (r = − 0.378, *P* < 0.001). **b** Correlation between serum adjusted-vitamin A concentrations and LOS in MPP children (r = − 0.384, P < 0.001)

To further evaluate the correlation between serum adjusted-vitamin A levels and fever duration as well as LOS, linear correlation tests were constructed in MPP children with adjusted-vitamin A measurements (*n* = 143). Results showed serum adjusted-vitamin A levels were negatively correlated with fever duration (Fig. 2a, r = − 0.378, *P* < 0.001) and LOS (Fig. 2b, r = − 0.384, P < 0.001).

### Correlation between vitamin a and BALF cytokine levels in MPP children

**Fig. 3:**
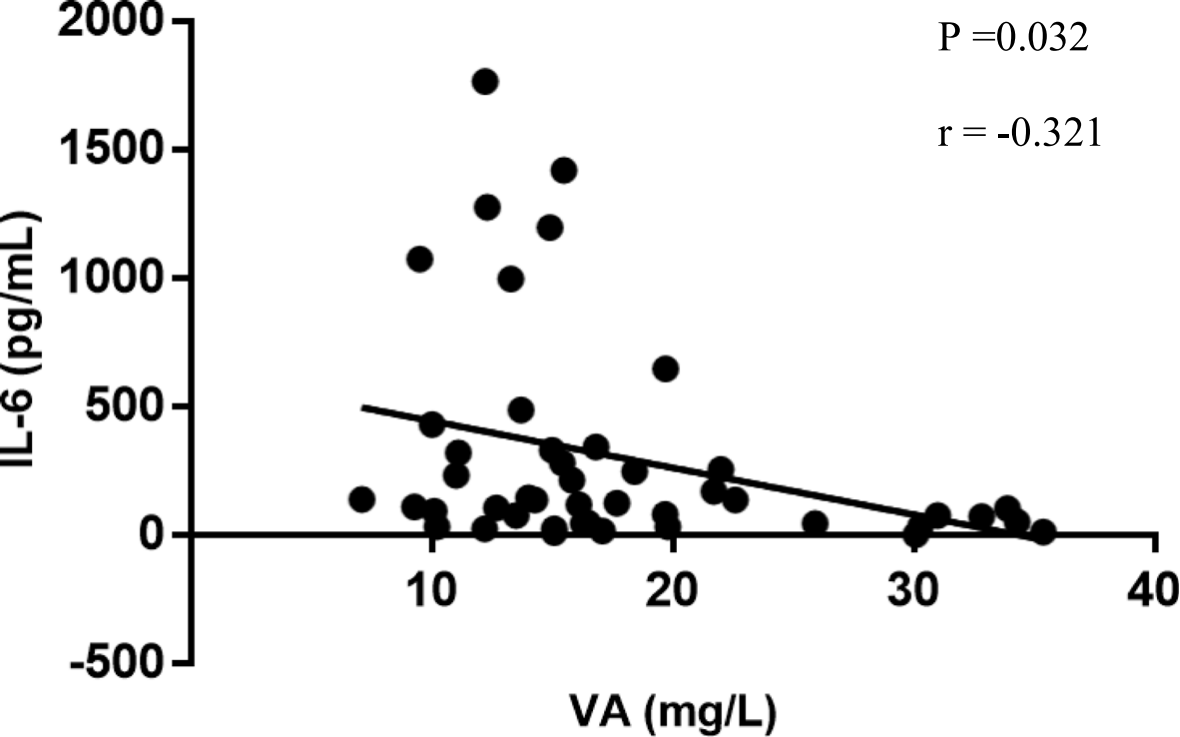
Correlation between serum adjusted-vitamin A levels and IL-6 concentrations in BALF in MPP children (r = − 0.321, *P* = 0.032)

To assess the correlation between adjusted-vitamin A and lung immunity, the linear correlation tests were applied for children who received bronchoalveolar lavage, results were shown in Fig. 3. We found a significantly negative correlation between adjusted-RBP and IL-6 levels (r = − 0.321, P = 0.032), while no significances were found with IL-2, IL-4, IL-10, TNF-α and IFN-γ.

## Discussion

*M. pneumoniae* is a leading cause of CAP, some MPP children could progress to RMPP. Researches have already noticed the important role of vitamin A in respiratory infection [22, 23], and emphasized accurate estimation of vitamin A deficiency [14]. Serum retinol or RBP concentrations provide vital information of vitamin A status and vitamin A deficiency (VAD) severity. Serum vitamin A concentrations could be affected by infection, for accurate estimation, adjusting it by CRP and/or AGP was recommended [14, 15]. As compared with other adjustment approaches, RC approach adjusting vitamin A in a continuous manner that better reflects the association between vitamin A and APPs. Thus, vitamin A concentrations were adjusted by CRP with RC approach in this study. To our knowledge, this is the first study to investigate the association between the real vitamin A status and RMPP incidence. We demonstrated serum vitamin A concentrations were significantly lower in RMPP children than those in GMPP patients. Insufficient serum vitamin A concentration was independently associated with RMPP incidence.

The overall incidence of RMPP in MPP patients was 16.02% in this study, which was similar with previous studies [24, 25]. However, we found the prevalence of VAD was 35.92% in this study, which was relatively lower than others’ reports [23, 26]. As serum vitamin A levels could be affected by infection [13], using vitamin A without adjusted by APPs could overestimate the prevalence of VAD in MPP children. The adjustments estimated VAD by mathematically removing or reducing the effect of elevated CRP in this study, which is important for decisions regarding nutrition interventions, programs, and policies.

Another important finding of our study is that sufficient serum vitamin A served as an independently protective factor for RMPP, every one unit decrease of adjusted-vitamin A (mg/L) resulted in 0.205 odds increase in RMPP incidence. Vitamin A is essential for the airway epithelium integrity [27], lung immune function and inflammation regulation [28], VAD may result in impaired mucosal barrier [29], disordered immune response [29, 30] and excessive cytokines release [31]. As we known, *M. pneumonia*e adhere to the host airway epithelium during MPP, followed by local airway epithelium damage and inflammatory cytokines release. Therefore, the decreased vitamin A during *M. pneumoniae* infection could deteriorate pulmonary injuries and clinical manifestations [32], which contributes to RMPP development together with longer fever duration and LOS. In malnourished children, vitamin A supplementation showed beneficial effects in acute lower respiratory infection (ALRI) children [23, 33, 34], those are evidences indicated the protective role of vitamin A in RMPP development. However, some studies indicated vitamin A supplementation in ALRI children had no benefits or modestly adverse effect in well-nourished children, which demonstrated the importance of accurate estimation of vitamin A status in planning and implementing interventions.

We also documented that one unit increase of CRP (mg/L) resulted in 0.05 odds increase in RMPP incidence, the median CRP concentrations in RMPP children were significantly higher than those in GMPP patients, which was in line with other studies [35, 36]. CRP is wildly known as a kind of acute phase protein, which rises rapidly and acutely in response to an inflammatory stimulus and reflect the individual immune response, translating into unfavorable conditions such as RMPP development. Meanwhile, prealbumin was found to be significantly lower in RMPP than GMPP patients, which correlated with RMPP incidence. Prealbumin is a carrier protein synthesized in the liver, it serves as an nonspecific host defense substance by eliminating toxic metabolites during infection [37]. Hrnciarikova [38] et al. found it negatively correlated with CRP and could serve as a negative acute phase protein, suggesting the reduction of prealbumin has similar significance with the increase of CRP in RMPP development.

In addition, we found LDH was significantly higher in RMPP children, univariate regression test also found its correlation with RMPP incidence [35, 39, 40]. It was confirmed that LDH elevated in many kinds of pulmonary diseases and reported to be associated with RMPP. LDH is released from cells after cell damage and can be used to monitor cell and tissue damage. Lung parenchymal cells, local inflammatory cells, including alveolar macrophages and neutrophils might be potential sources of LDH in pulmonary disorders [41, 42]. Thus, the higher level of LDH could translated into excessive inflammatory cell infiltration and severe lung injury, indicating increased RMPP incidence.

Besides, there were trends for correlations with ferritin and TNF-α, which agreed with other’ studies [43, 44]. In the linear correlation test, we found a significantly negative correlation between IL-6 and vitamin A. *M. pneumoniae* infections are closely related to stimulation of macrophages via toll-like receptors that release immunomodulatory and inflammatory cytokines and chemokines [33]. Ferritin is a kind of non-specific marker of inflammation induced by activated macrophages, which could also produce TNF-α [45] and interplay with IL-6 [46]. Thus, the increased level of ferritin and cytokines can reflect excessive inflammation and RMPP development. However, no significance was found between TNF-α and vitamin A in linear correlation analysis, this may relate to the small sample of bronchoalveolar lavage, which could underestimate the correlation between vitamin A and BALF cytokines.

There are potential limitations of this study. First, this is a single-center study, the data were collected from one academic teaching hospital in China, the results may not easily extrapolate to patients admitted to other regions. Second, the relatively small sample size of our study may reduce the ability to determine the statistical significance of the variables. Third, as the data were collected from the records retrospectively, some information was unfortunately missed, which may lead to imbalanced group sample size. A larger prospective study could help to evaluate the role of vitamin A for RMPP in different age groups, geographical locations.

## Conclusions

Serum vitamin A concentrations are independently associated with RMPP incidence, vitamin A levels may correlate with reduced incidence of RMPP.

## Data Availability

The data-sets analyzed during the current study are available from the corresponding author on reasonable request.

## Abbreviations

M. pneumoniae: Mycoplasma pneumoniae
CAP: Community-acquired pneumonia
MPP: M. pneumoniae pneumonia
RMPP: Refractory Mycoplasma pneumoniae pneumonia
LOS: Length of stay
APP: Acute phase protein
CRP: C-reactive protein
AGP: α-1-acid glycoprotein
VAD: Vitamin A deficiency
RBP: Retinol-binding protein
HPLC: High-performance liquid chromatography
GMPP: General *M. pneumoniae* pneumonia
PCR: Polymerase chain reaction
BMI: Body mass index
NPA: Nasopharyngeal aspirates
RC: Regression correction
BALF: Bronchoalveolar lavage fluid
CBA: Cytometric Bead Array
IQR: Interquartile range
OR: Odds ratio
CI: Confidence interval
LDH: Lactate dehydrogenase
